# Hypovitaminosis D and Metabolic Syndrome in Postmenopausal Women

**DOI:** 10.3390/healthcare10102026

**Published:** 2022-10-14

**Authors:** Haya Abuhijleh, Dana Alkhatib, Vijay Ganji

**Affiliations:** Human Nutrition Department, College of Health Sciences, QU Health, Qatar University, Doha P.O. Box 2713, Qatar

**Keywords:** 25-hydroxyvitamin D, hypovitaminosis D, metabolic syndrome, postmenopausal women, insulin resistance

## Abstract

Metabolic syndrome (MetSyn) is a precursor for several cardiometabolic diseases. The prevalence of MetSyn is higher in postmenopausal women compared to premenopausal women. The role of vitamin D in postmenopausal women is not clearly understood. Hypovitaminosis D is more prevalent in postmenopausal women compared to premenopausal women. For this review, Pubmed, Cochrane, SCOPUS, Embase, and Google Scholar databases were searched up to August 2022. Findings from one randomized controlled trial (RCT) and ten cross-sectional studies were included in this review. Several cross-sectional studies (8 out of 10 reviewed) unequivocally demonstrated an inverse association between serum 25-hydroxyvitamin D concentrations and MetSyn. However, RCTs are severely lacking in the effect of vitamin D intake on the biomarkers of MetSyn and the prevalence of MetSyn. Therefore, caution should be used in recommending mega doses of vitamin D supplements for postmenopausal women because of the potential adverse effects associated with this vitamer.

## 1. Introduction

Metabolic syndrome (MetSyn) or syndrome X is a global health concern [1] and is linked to chronic diseases such as obesity, type 2 diabetes mellitus (T2DM), and cardiovascular diseases (CVD) [2,3]. MetSyn is characterized by increased visceral adiposity, dyslipidemia (elevated triglycerides and low high-density lipoprotein (HDL) cholesterol), elevated blood pressure, and dysglycemia [4]. Additionally, MetSyn is associated with insulin resistance and elevated insulin concentrations [1]. Insulin resistance is linked to obesity and the risk of CVD, which in turn may contribute to dyslipidemia and perturbed glucose regulation [5]. Risk factors for MetSyn include older age, alcohol consumption, smoking, family history, and sedentary lifestyle [2]. The prevalence of MetSyn varies with the criteria used to define it. In the Asia-Pacific region, the prevalence of MetSyn in adults ranged from 20% to 25% [6]. However, in the same region, the prevalence of MetSyn in postmenopausal women ranged from 22.3% to 39.9% [7]. The prevalence of MetSyn in postmenopausal women varied from region to region: 29% in Puerto Rico, 31% in Iran, 48.9% in Brazil, and 55.5% in India [8]. The prevalence of MetSyn in postmenopausal women (57.8%) is much higher compared to premenopausal women (20%) [9].

The prevalence of vitamin D deficiency in the general population ranged between 20% and 90%, depending on the serum 25-hydroxyvitamin D (25(OH)D) cut-off values used to define vitamin D deficiency [10]. Because there are no consistent clinical guidelines for defining vitamin D deficiency, hypovitaminosis D has been defined as serum 25(OH)D concentrations of ˂30 nmol/L, ˂50 nmol/L, or ˂75 nmol/L [11,12,13]. Hypovitaminosis D prevalence varied by region: 44–96% in the Middle East and North Africa [14], 30–60% in Europe [15], and ~24% in the US [16]. Among several factors, dark skin tone [17] and adiposity [18] were directly related to the prevalence of vitamin D deficiency.

The Institute of Medicine (IOM) set the recommended dietary intake for vitamin D at 600 IU for adults up to 70 y old and 800 IU/d for people ˃70 y old [19]. On the other hand, the Endocrine Society (ES) recommended that people aged 19–70 y old and those aged more than 70 y old should consume at least 600 IU/d and 800 IU/d of vitamin D, respectively. Further, the ES recommended that vitamin D deficient adults take 50,000 IU/wk for 8 wk and then 1500–2000 IU/d for maintenance [13]. The tolerable upper intake level (UL) was set at 4000 IU/d for adults by the IOM and ES. Vitamin D’s classical role is to maintain the homeostasis of calcium and phosphorus and normal bone metabolism [20]. However, it has been recognized that vitamin D’s role goes beyond the bone.

Recent evidence links hypovitaminosis D to various non-communicable infirmities such as MetSyn, diabetes, cancer, and psychological disorders [21,22,23]. Eight studies included in this review found a higher prevalence of hypovitaminosis D among postmenopausal women compared to premenopausal women [24,25,26,27,28,29,30,31], while others did not find a significant association between the two variables [32,33,34]. Studies regarding vitamin D and MetSyn among postmenopausal women are still sparse and inconclusive [24,27,35], and reviews are lacking. Therefore, the goal of this review was to synthesize the current literature, particularly recent studies, about the relationship between hypovitaminosis D and MetSyn in postmenopausal women.

## 2. Methods

### 2.1. Studies Extraction Process

Several databases were searched to provide a reasonable breadth and depth of information on the topic. PubMed, Cochrane, SCOPUS, and Embase databases, were searched. Studies published until August 2022 were included in this review. However, all the studies were from the period 2011 to 2022 as there were no published reports before 2011. The following key terms were used in the search process: “vitamin D and metabolic syndrome and postmenopausal women; “25-hydroxyvitamin D and metabolic syndrome and postmenopausal women”; “25(OH)D and metabolic syndrome, and postmenopausal women”; and “hypovitaminosis D and metabolic syndrome and postmenopausal women”.

### 2.2. Studies Derivation

Based on this search, we identified a total of 63 records. Articles published in non-English languages were excluded (*n* = 3). Furthermore, 49 records were excluded for various reasons (duplicates, outcome measurements, study design, and did not fit inclusion criteria). Based on our search strategy, this narrative review was based on the results of ten cross-sectional studies and one randomized control trial (RCT). Menopausal status was defined as not having a menstrual cycle for ≥12 months. Serum 25(OH)D concentrations were used to assess vitamin D status in all studies. Because serum 25(OH) D has a longer half-life and represents both dietary vitamin D and endogenously synthesized vitamin D in the dermis, it is the most widely used biomarker of vitamin D status. In all studies (except one), the vitamin D deficiency was measured using serum 25(OH) D concentrations <50 nmol/L. In cross-sectional studies, the relationship between serum 25(OH)D and the prevalence of MetSyn in postmenopausal women was assessed with multivariate regression models. However, in the RCT, the postmenopausal women were randomized into two groups. The intervention group was given vitamin D3 at 1000 IU/d and the control group was given a placebo. The MetSyn components were assessed at baseline and endpoint for both groups.

## 3. Results of Studies on Hypovitaminosis D and MetSyn in Postmenopausal Women

In total, eleven studies investigated the association between serum vitamin D and MetSyn in post-menopause women (Table 1). These 11 studies formed the basis for this review.

### 3.1. RCT

One RCT found that postmenopausal women who consumed 1000 IU/d of vitamin D had improved markers of MetSyn compared to those women who took a placebo. In this RCT, 160 postmenopausal women were randomly allocated to either to the treatment group to take 1000 IU/d of vitamin D_3_ or to the placebo for 9 months [28]. In the multivariate-adjusted analysis, women supplemented with vitamin D_3_ had a lower risk of MetSyn (OR, 0.42; 95% CI, 0.21–0.83), hypertriglyceridemia (OR, 0.43; 95% CI, 0.22–0.85), and hyperglycemia (OR, 0.23; 95% CI, 0.10–0.52), compared to the placebo group. However, there was no change in blood pressure or anthropometric measures. [28].

### 3.2. Cross-Sectional Studies

In a cross-sectional study, postmenopausal women with hypovitaminosis D had a higher prevalence of MetSyn, hypertriglyceridemia, and low HDL-cholesterol than postmenopausal women with normal serum 25(OH)D [24]. In a study on 340 Thai postmenopausal women, hypovitaminosis D was found to increase the risk of MetSyn, hypertriglyceridemia, and obesity. Additionally, in this study, serum 25(OH)D was lower in postmenopausal women with MetSyn than in those without MetSyn [30]. Another study reported a relationship between serum 25(OH)D and MetSyn, as well as the risk factors associated with it, in Korean postmenopausal women [27]. Although no statistically significant associations were found between serum 25(OH)D concentrations and the prevalence of MetSyn (OR, 0.9; 95% CI, 0.76–1.05; *p* = 0.33), the ORs were significant for high blood pressure (OR, 0.83; 95% CI, 0.71–0.98), high serum triglycerides (OR, 0.83; 95% CI, 0.71–0.97), and reduced HDL-cholesterol (OR, 0.8; 95% CI, 0.69–0.93). Overall, women in the highest tertile of serum 25(OH)D had a significantly lower prevalence of elevated blood pressure (*p* = 0.02), elevated triglycerides (*p* = 0.014), and low HDL-cholesterol (*p* = 0.002) compared to those in the lowest tertile [27].

On the other hand, the association between serum 25(OH)D with various cardiometabolic risk factors and MetSyn was studied in 64 Indian postmenopausal women. Results showed no differences in cardiometabolic risk profiles between vitamin D deficient and sufficient women [36]. Both women with and without MetSyn had similar serum 25(OH)D. Insulin resistance was not evaluated in this study. This could have provided better insights into the possible link between the high prevalence of hypovitaminosis D and MetSyn. Another study investigated the relationship between serum 25(OH)D and vascular and bone health in postmenopausal women with MetSyn [33]. In this study, a high proportion of vitamin D deficient postmenopausal women had MetSyn but without a significant relation between serum 25(OH)D and vascular and bone health [33]. Although cross-sectional studies failed to show an association between serum 25(OH)D and endothelial functioning, a meta-analysis on RCTs have shown a positive effect of vitamin D supplementation on endothelial function, only in diabetic patients [35].

Huang et al. conducted a study on 616 Chinese postmenopausal women, and their results suggest a synergistic effect of vitamin D and estradiol deficiency on MetSyn in postmenopausal women [29]. There was a direct association between serum 25(OH)D and estradiol concentration. They observed that an increase in serum 25(OH)D was associated with improved blood pressure, lipid profile, and circulating glucose. After multivariable adjustment, the OR for MetSyn was 2.19 (95% CI, 1.19–4.01, *p* for trend, 0.009) for vitamin D deficient compared to sufficient women. After adjusting for estradiol concentrations, this association persisted [29]. Further, an association between vitamin D deficiency and insulin resistance was investigated in Saudi postmenopausal women (*n* = 300) with and without MetSyn [25]. Results revealed that the prevalence of increased waist circumference (WC), insulin resistance, and vitamin D deficiency was high in postmenopausal women. Recently, a study reported an inverse relationship between serum vitamin D concentrations and body adiposity markers such as BMI (*p* < 0.0005), WC (*p* < 0.044), fat mass (*p* < 0.003), android fat (*p* < 0.009), and gynecoid fat (*p* < 0.001) in postmenopausal Qatari women [38]. Using the data from the Korean National Health and Nutrition Examination Survey, investigators have reported that MetSyn markers and the MetSyn risk were statistically different in postmenopausal women between those who are vitamin D deficient and sufficient. Moreover, dietary consumption was found to modulate the effect of menopausal status and serum 25(OH)D on MetSyn risk and obesity, especially among postmenopausal women [37].

A recent quasi-experimental study reported a combination of aerobic exercise and 50,000 IU/d of vitamin D significantly reduced inflammatory markers such as C-reactive protein and interleukin-6 and improved all MetSyn markers in postmenopausal women [39]. Overall, evidence from cross-sectional studies suggest that, hypovitaminosis D is associated with the increased risk of developing MetSyn in postmenopausal women.

## 4. Discussion

### 4.1. Role of Vitamin D in MetSyn

Recent evidence has shown that vitamin D plays a role in various non-calcemic functions. Vitamin D receptors (VDR) have been mapped in a wide range of insulin-dependent cells and tissues (liver, skeletal muscle, and adipose tissues) [28], which implies that vitamin D plays a role in glucose utilization, insulin secretion, and insulin sensitivity. As a result, the potential derangements caused by vitamin D deficiency and the therapeutic potential of vitamin D have been the focus in recent times. During this time, the chronic disease burden has also increased. Overweight, obesity (especially abdominal obesity), and MetSyn were inversely related to serum 25(OH) D [25,26,27,28,29,30,40]. Hypovitaminosis D is linked to higher systolic blood pressure, lower HDL cholesterol, and insulin resistance [25,36,40,41]. Furthermore, lower serum 25(OH)D concentrations have been linked to increased morbidity and mortality related to myocardial infarction and diabetes [42,43,44,45]. Some of these negative effects could be reversed when vitamin D concentrations are normalized [46].

Vitamin D deficiency plays an important role in the pathogenesis of T2DM by increasing insulin resistance and promoting inflammation [41]. Polymorphism in the VDR genes has been associated with alterations in insulin secretion and sensitivity [47]. VDRs are found in insulin-secreting β-cells of the pancreas as well as 1 α-hydroxylase, which converts circulating 25(OH)D to active 1,25-dihydroxyvitamin D (1,25 (OH)_2_D) [46]. Previous studies have proposed an association between hypovitaminosis D and decreased peripheral insulin action, either through decreased expression of insulin receptors or through impairment in the downstream signaling of the insulin receptor [46]. Vitamin D stimulates the expression of the insulin receptor in peripheral tissues, increasing glucose uptake [48]. Additionally, because insulin-mediated intracellular processes are calcium-dependent, vitamin D status may have an indirect influence on insulin sensitivity in skeletal muscles and adipose tissues [28,48].

An inverse association was observed between serum 25(OH)D and triglyceride concentrations in postmenopausal women [49]. This inverse association between vitamin D and serum lipids could be due to a decrease in intestinal absorption and synthesis of lipids and decreased lipolysis [24,49]. A meta-analysis of randomized controlled trials reported a positive association between serum 25(OH)D concentrations and HDL cholesterol, suggesting that hypovitaminosis D may contribute to an atherogenic lipid profile, a major risk factor for the development of coronary artery disease [50]. A direct association between serum 25(OH)D and apolipoprotein A-1 suggests a possible role of vitamin D in the formation of HDL particles in blood [50,51]. Moreover, it has been proposed that VDRs regulate cholesterol concentrations by upregulating the synthesis of bile acids from cholesterol [24]. Vitamin D influences lipid metabolism by inhibiting adipogenic transcription factors and lipid accumulation during adipocyte differentiation. Metabolites of vitamin D induce adipokine production and inflammatory response in adipose tissue [52], which results in an impairment in the normal metabolic function of adipose tissue in hypovitaminosis D.

Because adipose tissue plays a role in energy balance, lipid metabolism, and inflammation, serum 25(OH)D can have a major impact on metabolic health maintenance [53]. Several epidemiological studies have shown an inverse association between serum 25(OH)D and obesity markers such as BMI and WC [54]. This association was likely due to the sequestration of vitamin D in adipose tissue, leading to lower serum 25(OH)D [52,54]. As previously stated, dietary intake can influence the effect of vitamin D status on MetSyn risk. It has been reported that postmenopausal women tend to have low dietary protein intake, an increased prevalence of obesity, and an increased risk of MetSyn [55]. The metabolic pathologies that are commonly seen in postmenopausal women, cannot be solely attributed to vitamin D deficiency alone. The postmenopausal women group were older and generally engaged in less physical activity, leading to decreased sun exposure, which made them more vitamin D deficient. Additionally, decreased physical activity is related to a higher risk of obesity. Due to hormonal changes related to aging, postmenopausal women tend to have unfavorable biomarkers of body composition, i.e., increased body adiposity and decreased lean body mass. High protein diets (which tend to be low in carbohydrates) have been linked to reduced body weight, fat mass, triglycerides, and blood pressure, which are components of MetSyn [37,56,57].

### 4.2. Serum Vitamin D, Estrogen, and MetSyn

**Figure 1:**
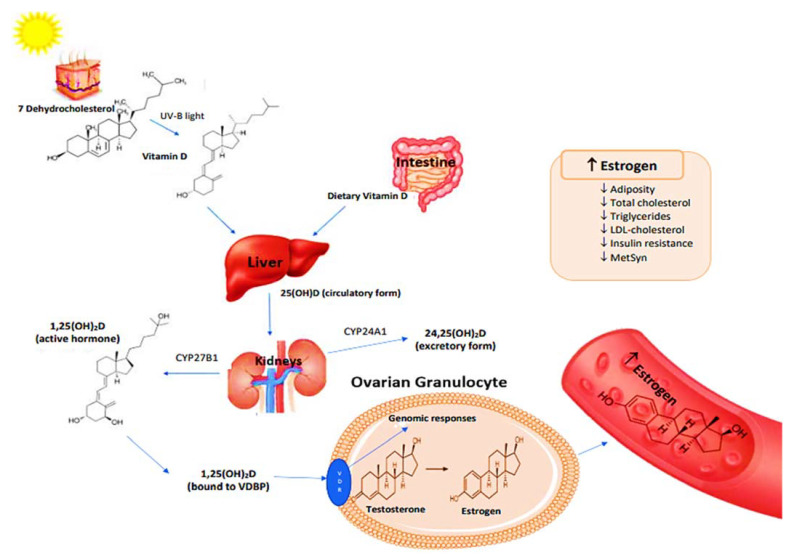
Metabolism of vitamin D-activation and genomic functions [58,59]. Abbreviations: 1,25(OH)2D, 1,25-dihydroxyvitamin D; 25(OH)D, 25-hydroxyvitamin D; 24,25(OH)2D, 24,25-dihydroxyvitamin D; CYP24A1, cytochrome P450 family 24 subfamily A member 1 or 24-hydroxylase; CYP27B1, cytochrome P450 family 27 subfamily B member 1 or 1α-hydroxylase, LDL, low-density-lipoprotein; MetSyn, metabolic syndrome.

A brief role of vitamin D in genomic function is described in Figure 1 [58,59]. The binding of 1,25(OH)_2_D to VDRs triggers the genetic expression of enzymes that are responsible for converting the androgens and estrogens into their active forms [58]. One such enzyme is 17β-hydroxysteroid dehydrogenase, which regulates the concentration of intracellular steroidal hormones in target tissues [58]. Aromatase, which is responsible for estrogen synthesis, is an enzyme that has several functions in different tissues such as the ovaries, breasts, and adipose tissues [59]. In tissues such as ovarian granulocytes, 1,25(OH)_2_D modifies genes that express aromatase [58]. It has also been found that vitamin D deficiency is linked to various reproductive disorders in women, such as subfertility, polycystic ovarian syndrome, and endometriosis [59]. Therefore, it is reasonable to assume that hypovitaminosis D in postmenopausal women can exacerbate the pathologies of MetSyn. It is unclear whether increased 25(OH)D can reduce MetSyn biomarkers and the risk for heart diseases in postmenopausal women.

## 5. Strengths and Limitations

The reviewed studies have a few strengths. A few studies had a large sample size, and the results can be applied to the sample population at large. One study excluded women who had a hysterectomy to reduce the bias of having participants with an inaccurate status of menopause. Cross-sectional studies have considered several confounding variables that are known to affect serum vitamin D concentrations. Further, there is a need for longitudinal studies and RCTs, to understand the association between MetSyn components and hypovitaminosis D, in postmenopausal women. The majority of the studies reviewed (10 out of 11) are cross-sectional studies. In these studies, causality should not be assumed. The limitations of the reviewed studies are inconsistencies between studies in terms of MetSyn components cutoff points and lack of adjustment for certain factors that might affect the serum vitamin D concentrations. Cross-sectional studies did not take sunlight exposure into account as sunlight is one of the important contributors to vitamin D status. However, it is not known how the lack of adjustment for sunlight exposure affected the relationship between serum vitamin D and MetS in postmenopausal women. In cross-sectional studies, the vitamin D assessment was based on a single serum vitamin D measurement. A single measurement of serum vitamin D may not be representative of their normal vitamin D concentrations. Results from studies with small sample sizes cannot be generalized to the general population. The studies reviewed are heterogeneous in nature. They differ in the way vitamin D deficiency, insufficiency, and sufficiency were categorized. Studies are conducted in different countries with varied sample sizes and environments. Therefore, the findings from these studies may not be generalizable to postmenopausal women globally.

## 6. Recommendations

An emphasis should be placed on improving the vitamin D status in postmenopausal women by advocating the consumption of fish and vitamin D fortified foods. Because food sources of vitamin D are limited, it is tempting to reach out to supplements to boost vitamin D intake. Although vitamin D safety is well established in lower doses, the safety of vitamin D at higher doses is not known. As a result, postmenopausal women should be advised to consult health care professionals before they start consuming vitamin D supplements containing more than 4000 IU/d (UL), although a dosage level up to 10,000 IU/d is considered safe by many health experts. Caution should be used in recommending large doses of vitamin D due to potential health risks associated with vitamin D, although rare, such as kidney stones, calcification of soft tissues, hypercalcemia, gastrointestinal symptoms, altered mental status, and elevated blood pressure. Because of these potential adverse effects, the approach to treating vitamin D deficiency should be under medical surveillance only.

## 7. Conclusions

Evidence is accumulating on the association between serum 25(OH)D and MetSyn in postmenopausal women. Epidemiological evidence linking hypovitaminosis D with MetSyn and its pathologies is strong. The majority of the studies showed an inverse association between serum 25(OH)D and MetSyn in postmenopausal women. There are no data to suggest the optimum serum 25(OH)D concentration or dietary vitamin D intake levels at which health benefits accrue in postmenopausal women. There is a need for controlled studies to test to what level an improved vitamin D status can lead to reduced pathologies associated with MetSyn in postmenopausal women. Further, the exact mechanism through which vitamin D acts to ameliorate the pathologies associated with MetSyn needs further clarification in this population.

## Figures and Tables

**Table 1:** Summary of studies on serum 25(OH)D concentrations and MetSyn in postmenopausal women ^1^.

Reference and Location	Age, y (Mean ± SD or Range)	Study Design and *n*	Intervention	MetSyn Criteria	Assessment of Serum 25(OH)D (nmol/L)	Findings and Conclusions ^2^
Ferreira et al., 2020, Brazil [28]	58.8 ± 6.6	RCT PMW, *n* = 160 Treatment group, *n* = 80 Control group, *n* = 80 9 month	Treatment group: Vitamin D supplementation, 1000 IU/d Control group: Placebo	Women meeting ≥ 3 of the following: WC > 88 cm TG ≥ 150 mg/dL HDL-C < 50 mg/dL BP ≥ 130/85 mmHg FBG ≥ 100 mg/dL	Sufficient, ≥75 Insufficient, 50–75 Deficient, <50	Vitamin D supplementation: ↓TG ↓HOMA-IR ↓Risk of MetSyn ↓Risk of hypertriglyceridemia ↓Risk of hyperglycemia
Schmitt et al., 2018, Brazil [24]	45–75	Cross-sectional PMW, *n* = 463 Vitamin D sufficient, *n* = 148 Vitamin D insufficient, *n* = 151 Vitamin D deficient, *n* = 164	No intervention	Women meeting ≥ 3 of the following: WC > 88 cm TG ≥ 150 mg/dL HDL-C < 50 mg/dL BP ≥ 130/85 mmHg FBG ≥ 100 mg/dL	Sufficient, ≥75 Insufficient, 50–75 Deficient, <50	Vitamin D insufficiency and deficiency were associated with: ↑MetSyn ↑TG ↑TC ↑HOMA-IR ↓HDL-C
Jeenduan, et al., 2020, Thailand [30]	62.7 ± 9.8	Cross-sectional PMW, *n* = 340 Hypovitaminosis D (insufficient and deficient) *n* = 194 Vitamin D sufficient, *n* = 146	No intervention	Women meeting ≥ 3 of the following: WC ≥ 80 cm TG ≥ 150 mg/dL HDL-C < 50 mg/dL BP ≥ 130/85 mmHg FBG ≥ 100 mg/dL	Sufficient, ≥75 Insufficient, 50–75 Deficient, <50	Low serum 25(OH)D was associated with: ↑MetSyn ↑TG ↑TC ↑WC
Chon et al., 2014,Korea [27]	<50–>70	Cross-sectional PMW, *n* = 4364 Vitamin D tertile 1, *n* = 1454 Vitamin D tertile 2, *n* = 1456 Vitamin D tertile 3, *n* = 1454	No intervention	Women meeting ≥ 3 of the following: WC ≥ 85 cm TG ≥ 150 mg/dL HDL-C < 50 mg/dL BP ≥ 130/85 mmHg FBG ≥ 100 mg/dL	Sufficient, ≥75 Insufficient, 50–75 Deficient, <50	Vitamin D sufficiency was associated with: ↓BP ↓TG ↑HDL-C
Mitra et al., 2016,India [36]	51 ± 7.75	Cross-sectional PMW, *n* = 64 Vitamin D sufficient, *n* = 16 Vitamin D insufficient, *n* = 15 Vitamin D deficient, *n* = 33	No intervention	Women meeting ≥ 3 of the following: WC ≥ 80 cm TG ≥ 150 mg/dL HDL-C < 50 mg/dL BP ≥ 130/8 5 mmHg FBG ≥ 100 mg/dL	Sufficient, >75 Insufficient, 52.5–72.5 Deficient, <50	No relation between serum 25(OH)D and cardiometabolic risk factors
Srimani et al., 2017, Korea [31]	45–70	Cross-sectional PMW, *n* = 222 Vitamin D sufficient, *n* = 67 Vitamin D insufficient, *n* = 42 Vitamin D deficient, *n* = 113	No intervention	Women meeting ≥ 3 of the following: WC ≥ 80 cm TG ≥ 150 mg/dL HDL-C < 50 mg/dL BP ≥ 130/85 mmHg FBG ≥ 110 mg/dL	Sufficient, 75–100 Insufficient, 50–75 Deficient, <50	Serum 25(OH)D was associated with: ↑WC ↑FBG ↑TG ↑BP
Dadonienėet al., 2018, Lithuania [33]	57.9 ± 3.9	Cross-sectional PMW, *n* = 210 Vitamin D sufficient, *n* = 84 Vitamin D deficient, *n* = 126	No intervention	Women meeting ≥ 3 of the following: WC > 88 cm TG ≥ 150 mg/dL HDL-C < 50 mg/dL BP ≥ 130/85 mmHg FBG ≥ 100 mg/dL	Sufficient, ≥50 Deficient, <50	No significant relation between serum 25(OH)D and vascular stiffness
Chacko et al., 2011, USA [26]	63.3 ± 7.5	Cross-sectional/Case-control PMW, *n* = 292 Vitamin D tertile 1, *n* = 96 Vitamin D tertile 2, *n* = 94 Vitamin D tertile 3, *n* = 102	No intervention **Cases:** 1 g calcium carbonate and 400 IU vitamin D_3_ **Controls:** Placebo	Women meeting ≥ 3 of the following: WC > 88 cm TG ≥ 150 mg/dL HDL-C < 50 mg/dL BP ≥ 130/85 mmHg FBG ≥ 100 mg/dL	1st tertile, 26 2nd tertile, 43 3rd tertile, 70	Serum 25(OH)D was associated with: ↓Adiposity ↓TG ↓TG to HDL-C ratio ↓MetSyn
Alissa et al., 2001, Saudi Arabia [25]	46–88	Cross-sectional PMW, *n* = 300 Vitamin D insufficient and sufficient, *n* = 201 Vitamin D deficient, *n* = 99	No intervention	Women with abdominal obesity (WC > 88 cm) with ≥2: TG ≥ 150 mg/dL HDL-C < 40 mg/dL BP ≥ 130/85 mmHg FBG ≥ 110 mg/dL	Insufficient and sufficient, ≥50 Deficient, <50	Serum 25(OH)D was associated with: ↓TG ↓FBG ↓DBP
Huang et al., 2019, China [29]	63.7 ± 7.7	Cross-sectional PMW, *n* = 616 Vitamin D sufficient, *n* = 192 Vitamin D insufficient, *n* = 312 Vitamin D deficient, *n* = 112	No Intervention	Women meeting ≥ 3 of the following: WC ≥ 80 cm TG ≥ 150 mg/dL HDL-C < 50 mg/dL BP ≥ 130/85 mmHg FBG ≥ 100 mg/dL	Sufficient, >75 Insufficient, 50–75 Deficient, <50	Serum 25(OH)D was associated with: ↑Estradiol ↓lipid profile ↓TC ↓TG ↓BP ↓FBG ↓MetSyn
Chun et al., 2020, Korea [37]	46.1	Cross-sectional PMW, *n* = 8326 Sufficient, *n* = 1996 Insufficient, *n* = 6330	No intervention	Women meeting ≥ 3 of the following: WC > 85 cm TG ≥ 150 mg/dL HDL-C < 50 mg/dL BP ≥ 130/85 mmHg FBG ≥ 100 mg/dL	Sufficient, ≥50 Deficient, <50	PMW with deficient vitamin D had: ↑BMI ↑WC ↑FBG ↑TG ↓HDL-C

^1^ Abbreviations: 25(OH)D, 25-hydroxyvitamin D; BMI, body mass index; BP, blood pressure; DBP, diastolic blood pressure; FBG, fasting blood glucose; HDL-C, high-density lipoprotein cholesterol; HOMA-IR, homeostatic model assessment-insulin resistance; IU, international unit/s; MetSyn, metabolic syndrome; PMW, postmenopausal women; RCT, randomized controlled trials; SD, standard deviation; TC, total cholesterol; TG, triglycerides; WC, waist circumference. ^2^ ↑ = increased; ↓ = decreased.

## Data Availability

Not applicable.

## References

[1] Anastasaki M., Papadakis S., Linardakis M., Anyfantakis D., Symvoulakis E.K., Lionis C., on behalf of the Cretan Primary Care Research Group (2020). Burden of metabolic syndrome among primary care patients in Crete, Greece: A descriptive study. Eur. J. Gen. Pract..

[2] Belete R., Ataro Z., Abdu A., Sheleme M. (2021). Global prevalence of metabolic syndrome among patients with type I diabetes mellitus: A systematic review and meta-analysis. Diabetol. Metab. Syndr..

[3] McCracken E., Monaghan M., Sreenivasan S. (2018). Pathophysiology of the metabolic syndrome. Clin. Dermatol..

[4] Detection, N.C.E.P.E.P.o. and T.o.H.B.C.i. Third Report of the National Cholesterol Education Program (NCEP) Expert Panel on Detection, Evaluation, and Treatment of High Blood Cholesterol in Adults (Adult Treatment Panel III), Adults 2002.

[5] Zhang R., Dong S.Y., Wang F., Ma C., Zhao X.L., Zeng Q., Fei A. (2018). Associations between body composition indices and metabolic disorders in Chinese adults: A cross-sectional observational study. Chin. Med. J..

[6] International Diabetes Federation (2006). The IDF Consensus Worldwide Definition of the Metabolic Syndrome. http://www.idf.org/webdata/docs/IDF_Meta_def_final.pdf.

[7] Kwon H.S., Park Y.M., Lee H.J., Lee J.H., Choi Y.H., Ko S.H., Lee J.M., Kim S.R., Kang S.Y., Lee W.C. (2005). Prevalence and clinical characteristics of the metabolic syndrome in middle-aged Korean adults. Korean J. Intern. Med..

[8] Marchi R.D., Dell’Agnolo C.M., Lopes T.C.R., Gravena A.A.F., Demitto M.D.O., Brischiliari S.C.R., Borghesan D.H.P., de Barros Carvalho M.D., Pelloso S.M. (2017). Prevalence of metabolic syndrome in pre-and postmenopausal women. Arch. Endocrinol. Metab..

[9] Sapkota A.S., Sapkota A., Acharya K., Raut M., Jha B. (2015). Study of metabolic syndrome in postmenopausal women. Ann. Clin. Chem. Lab. Med..

[10] Yeh Y.C., Chen K.W., Chen C.W., Yuan K.C., Wang I., Hung F.M., Wang A.Y., Wang Y.C., Kuo Y.T., Kuo L.K. (2021). Prevalence of vitamin D deficiency and associated factors in critically ill patients: A multicenter observational study. Front. Nutr..

[11] Camacho P.M., Petak S.M., Binkley N., Clarke B.L., Harris S.T., Hurley D.L., Kleerekoper M., Lewiecki E.M., Miller P.D., Watts N.B. (2016). American Association Of Clinical Endocrinologists and American College of Endocrinology clinical practice guidelines for the diagnosis and treatment of postmenopausal osteoporosis—2016--executive summary. Endocr. Pract..

[12] Del Valle H.B., Yaktine A.L., Taylor C.L., Ross A.C. (2011). Dietary Reference Intakes for Calcium and Vitamin D.

[13] Holick M.F., Binkley N.C., Bischoff-Ferrari H.A., Gordon C.M., Hanley D.A., Heaney R.P., Murad M.H., Weaver C.M., Endocrine Society (2011). Evaluation, Treatment, and Prevention of Vitamin D Deficiency: An Endocrine Society Clinical Practice Guideline. J. Clin. Endocrinol. Metab..

[14] Chakhtoura M., Rahme M., Chamoun N., Fuleihan G.E.-H. (2018). Vitamin D in the Middle East and North Africa. Bone Rep..

[15] Lips P., Cashman K.D., Lamberg-Allardt C., Bischoff-Ferrari H.A., Obermayer-Pietsch B., Bianchi M.L., Stepan J., El-Hajj Fuleihan G., Bouillon R. (2019). Current vitamin D status in European and Middle East countries and strategies to prevent vitamin D deficiency: A position statement of the European Calcified Tissue Society. Eur. J. Endocrinol..

[16] Amrein K., Scherkl M., Hoffmann M., Neuwersch-Sommeregger S., Köstenberger M., Berisha A.T., Martucci G., Pilz S., Malle O. (2020). Vitamin D deficiency 2.0: An update on the current status worldwide. Eur. J. Clin. Nutr..

[17] Dong H., Asmolovaite V., Marseal N., Mearbon M. (2021). Vitamin D status and dietary intake in young university students in the UK. Nutr. Food Sci..

[18] González-Molero I., Rojo-Martínez G., Morcillo S., Gutierrez C., Rubio E., Pérez-Valero V., Esteva I., De Adana M.S.R., Almaraz M.C., Colomo N. (2013). Hypovitaminosis D and incidence of obesity: A prospective study. Eur. J. Clin. Nutr..

[19] Ross A.C., Taylor C.L., Yaktine A.L., Del Valle H.B. (2011). Institute of Medicine (US) Committee to Review Dietary Reference Intakes for Vitamin D and Calcium. Dietary Reference Intakes for Calcium and Vitamin D.

[20] Gil Hernández Á., Plaza Díaz J., Mesa García M.D. (2018). Vitamin D: Classic and Novel Actions. Ann. Nutr. Metab..

[21] Ganji V., Tangpricha V., Zhang X. (2020). Serum vitamin D concentration≥ 75 nmol/L is related to decreased cardiometabolic and inflammatory biomarkers, metabolic syndrome, and diabetes; and increased cardiorespiratory fitness in US adults. Nutrients.

[22] Ganji V., Sukik L., Hoque B., Boutefnouchet L., Shi Z. (2022). Association of Serum 25-Hydroxyvitamin D Concentration with Breast Cancer Risk in Postmenopausal Women in the US. J. Pers. Med..

[23] Tehrani A.N., Farhadnejad H., Salehpour A., Hekmatdoost A. (2020). Vitamin D intake and risk of psychological disorders among female adolescents. Nutr. Food Sci..

[24] Schmitt E.B., Nahas-Neto J., Dias F.N.B., Poloni P.F., Orsatti C., Nahas E.A.P. (2018). Vitamin D deficiency is associated with metabolic syndrome in postmenopausal women. Maturitas.

[25] Alissa E.M., Alnahdi W.A., Alama N., Ferns G.A. (2014). Insulin resistance in Saudi postmenopausal women with and without metabolic syndrome and its association with vitamin D deficiency. J. Clin. Transl. Endocrinol..

[26] Chacko S.A., Song Y., Manson J.E., Van Horn L., Eaton C., Martin L.W., McTiernan A., Curb J.D., Wylie-Rosett J., Phillips L.S. (2011). Serum 25-hydroxyvitamin D concentrations in relation to cardiometabolic risk factors and metabolic syndrome in postmenopausal women. Am. J. Clin. Nutr..

[27] Chon S.J., Yun B.H., Jung Y.S., Cho S., Choi Y.S., Kim S.Y., Lee B.S., Seo S.K. (2014). Association between Vitamin D Status and Risk of Metabolic Syndrome among Korean Postmenopausal Women. PLoS ONE.

[28] Ferreira P.P., Cangussu L., Bueloni-Dias F.N., Orsatti C., Schmitt E.B., Nahas-Neto J., Nahas E.A.P. (2019). Vitamin D supplementation improves the metabolic syndrome risk profile in postmenopausal women. Climacteric.

[29] Huang H., Guo J., Chen Q., Chen X., Yang Y., Zhang W., Liu Y., Chen X., Yang D. (2019). The synergistic effects of vitamin D and estradiol deficiency on metabolic syndrome in Chinese postmenopausal women. Menopause.

[30] Jeenduang N., Plyduang T., Horpet D. (2020). Association of 25-hydroxyvitamin D levels and metabolic syndrome in Thai postmenopausal women. Diabetes Metab. Syndr. Clin. Res. Rev..

[31] Srimani S., Saha I., Chaudhuri D. (2017). Prevalence and association of metabolic syndrome and vitamin D deficiency among postmenopausal women in a rural block of West Bengal, India. PLoS ONE.

[32] Andreozzi P., Verrusio W., Viscogliosi G., Summa M.L., Gueli N., Cacciafesta M., Albanese C.V. (2015). Relationship between vitamin D and body fat distribution evaluated by DXA in postmenopausal women. Nutrition.

[33] Dadoniene J., Cypiene A., Rinkuniene E., Badariene J., Laucevicius A. (2018). Vitamin D, cardiovascular and bone health in postmenopausal women with metabolic syndrome. Adv. Clin. Exp. Med..

[34] Moghassemi S., Marjani A. (2014). The effect of short-term vitamin D supplementation on lipid profile and blood pressure in post-menopausal women: A randomized controlled trial. Iran. J. Nurs. Midwifery Res..

[35] Hussin A.M., Ashor A.W., Schoenmakers I., Hill T., Mathers J.C., Siervo M. (2017). Effects of vitamin D supplementation on endothelial function: A systematic review and meta-analysis of randomised clinical trials. Eur. J. Nutr..

[36] Mitra S. (2016). Vitamin D Status and Cardio-Metabolic Risk in Indian Postmenopausal Women. J. Clin. Diagn. Res..

[37] Chun H., Kim G.D., Doo M. (2020). Differences in the association among the vitamin D concentration, dietary macronutrient consumption, and metabolic syndrome depending on pre-and postmenopausal status in Korean women: A cross-sectional study. Diabetes Metab. Syndr. Obes. Targets Therapy.

[38] Ganji V., Shi Z., Alshami H., Ajina S., Albakri S., Jasim Z. (2021). Serum 25-hydroxyvitamin D concentrations are inversely associated with body adiposity measurements but the association with bone mass is non-linear in postmenopausal women. J. Steroid Biochem. Mol. Biol..

[39] Nazarabadi P.N., Etemad Z., Hoseini R., Moradi F. (2022). Anti-Inflammatory Effects of a Period of Aerobic Training and Vitamin D Supplementation in Postmenopausal Women with Metabolic Syndrome. Int. J. Prev. Med..

[40] Lee S., Kim S.M., Park H., Choi K., Cho G., Ko B., Kim J. (2013). Serum 25-hydroxyvitamin D levels, obesity and the metabolic syndrome among Korean children. Nutr. Metab. Cardiovasc. Dis..

[41] Wang W., Zhang J., Wang H., Wang X., Liu S. (2019). Vitamin D deficiency enhances insulin resistance by promoting inflammation in type 2 diabetes. Int. J. Clin. Exp. Pathol..

[42] Saliba W., Barnett O., Rennert H.S., Rennert G. (2012). The Risk of All-Cause Mortality Is Inversely Related to Serum 25(OH)D Levels. J. Clin. Endocrinol. Metab..

[43] Sofianopoulou E., Kaptoge S.K., Afzal S., Jiang T., Gill D., Gundersen T.E., Bolton T.R., Allara E., Arnold M.G., Mason A.M. (2021). Estimating dose-response relationships for vitamin D with coronary heart disease, stroke, and all-cause mortality: Observational and Mendelian randomisation analyses. Lancet Diabetes Endocrinol..

[44] Thomas G.N., ó Hartaigh B., Bosch J.A., Pilz S., Loerbroks A., Kleber M.E., Fischer J.E., Grammer T.B., Böhm B.O., & März W. (2012). Vitamin D levels predict all-cause and cardiovascular disease mortality in subjects with the metabolic syndrome: The Ludwigshafen Risk and Cardiovascular Health (LURIC) Study. Diabetes Care.

[45] Wan Z., Guo J., Pan A., Chen C., Liu L., Liu G. (2020). Association of Serum 25-Hydroxyvitamin D Concentrations With All-Cause and Cause-Specific Mortality Among Individuals With Diabetes. Diabetes Care.

[46] Wimalawansa S.J. (2018). Associations of vitamin D with insulin resistance, obesity, type 2 diabetes, and metabolic syndrome. J. Steroid Biochem. Mol. Biol..

[47] Lips P., Eekhoff M., van Schoor N., Oosterwerff M., de Jongh R., Krul-Poel Y., Simsek S. (2017). Vitamin D and type 2 diabetes. J. Steroid Biochem. Mol. Biol..

[48] Yaribeygi H., Maleki M., Sathyapalan T., Iranpanah H., Orafai H.M., Jamialahmadi T., Sahebkar A. (2020). The molecular mechanisms by which vitamin D improve glucose homeostasis: A mechanistic review. Life Sci..

[49] Jorde R., Grimnes G. (2011). Vitamin D and metabolic health with special reference to the effect of vitamin D on serum lipids. Prog. Lipid Res..

[50] Wang H., Xia N., Yang Y., Peng D.-Q. (2012). Influence of vitamin D supplementation on plasma lipid profiles: A meta-analysis of randomized controlled trials. Lipids Health Dis..

[51] Kazlauskaite R., Powell L.H., Mandapakala C., Cursio J.F., Avery E.F., Calvin J. (2010). Vitamin D is associated with atheroprotective high-density lipoprotein profile in postmenopausal women. J. Clin. Lipidol..

[52] Hajri T., Hall A.M., Jensen D.R., Pietka T.A., Drover V.A., Tao H., Eckel R., Abumrad N.A. (2007). CD36-Facilitated Fatty Acid Uptake Inhibits Leptin Production and Signaling in Adipose Tissue. Diabetes.

[53] Vimaleswaran K.S., Berry D.J., Lu C., Tikkanen E., Pilz S., Hiraki L.T., Cooper J.D., Dastani Z., Li R., Houston D.K. (2013). Causal Relationship between Obesity and Vitamin D Status: Bi-Directional Mendelian Randomization Analysis of Multiple Cohorts. PLoS Med..

[54] Taheri E., Saedisomeolia A., Djalali M., Qorbani M., Civi M.M. (2012). The relationship between serum 25-hydroxy vitamin D concentration and obesity in type 2 diabetic patients and healthy subjects. J. Diabetes Metab. Disord..

[55] Ko S.-H., Kim H.-S. (2020). Menopause-Associated Lipid Metabolic Disorders and Foods Beneficial for Postmenopausal Women. Nutrients.

[56] Clifton P., Condo D., Keogh J. (2014). Long term weight maintenance after advice to consume low carbohydrate, higher protein diets—A systematic review and meta analysis. Nutr. Metab. Cardiovasc. Dis..

[57] Santesso N., Akl E.A., Bianchi M., Mente A., Mustafa R., Heels-Ansdell D., Schunemann H.J. (2012). Effects of higher- versus lower-protein diets on health outcomes: A systematic review and meta-analysis. Eur. J. Clin. Nutr..

[58] Shahrokhi S.Z., Ghaffari F., Kazerouni F. (2015). Role of vitamin D in female Reproduction. Clin. Chim. Acta.

[59] Voulgaris N., Papanastasiou L., Piaditis G., Angelousi A., Kaltsas G., Mastorakos G., Kassi E. (2017). Vitamin D and aspects of female fertility. Hormones.

